# *In vivo* assessment of human brainstem cerebrovascular function: a multi-inversion time pulsed arterial spin labelling study

**DOI:** 10.1038/jcbfm.2014.39

**Published:** 2014-03-05

**Authors:** Esther AH Warnert, Ashley D Harris, Kevin Murphy, Neeraj Saxena, Neeta Tailor, Nigel S Jenkins, Judith E Hall, Richard G Wise

**Affiliations:** 1 Cardiff University Brain Research and Imaging Centre (CUBRIC), School of Psychology, Cardiff University, Cardiff, UK; 2 Russell H. Morgan Department of Radiology and Radiological Science, The Johns Hopkins University, Baltimore, Maryland, USA; 3 F.M. Kirby Center for Functional Brain Imaging, Kennedy Krieger Institute, Baltimore, Maryland, USA; 4 Department of Anaesthetics and Intensive Care Medicine, School of Medicine, Cardiff University, Cardiff, UK

**Keywords:** arterial spin labelling, brainstem, cerebral hemodynamics

## Abstract

The brainstem (BS) is involved in critical physiologic processes, including control of cardiovascular and respiratory functions. This study implements a multi-inversion time pulsed arterial spin labelling (MTI PASL) imaging sequence that addresses the challenges of BS imaging and aims to measure normal and elevated BS perfusion in healthy volunteers. An initial experiment was performed to obtain the kinetic curve of the label in the BS and consequently to estimate the label arrival times and tissue perfusion in seven participants. A second experiment estimated the BS cerebral vascular reactivity (CVR) to hypercapnia in 10 participants. Images were acquired with a gradient-echo sequence with two spiral interleaves and short echo time (TE=2.7 ms). Data were analyzed with a two-compartment model, including a tissue and arterial component. In both experiments, perfusion in the BS was significantly lower than in cortical gray matter (repeated measures analysis of variance (RM-ANOVA), *P*<0.05), which is as expected since the BS consists of gray and white matter, the latter typically showing lower perfusion. The BS CVR found here is comparable to previous reports obtained with positron emission tomography (PET) imaging. Multi-inversion time pulsed ASL in combination with a two-compartment signal model can be used to assess BS perfusion and CVR.

## Introduction

The brainstem (BS) has a key role in many physiologic processes central to the survival of the organism. Recent functional magnetic resonance imaging studies have investigated the involvement of BS nuclei in the control of cardiovascular function,^1^ respiration,^2^ pain processing,^3^ and arousal.^4^ Brainstem pathology can be life-threatening or can lead to severe disability. For example, BS ischemia can lead to the locked-in syndrome.^5^

Positron emission tomography (PET) has been used in clinical studies of BS pathologies^6, 7^ to assess cerebral glucose metabolism and/or cerebral blood flow (CBF). However, PET requires the administration of a radioactive tracer. Magnetic resonance imaging-based arterial spin labelling (ASL) offers a noninvasive way to measure tissue perfusion in which the water in arterial blood is radio-frequency labelled as it flows through major brain-feeding arteries before the labelled arterial blood enters the brain tissue.^8^ Currently, there is a limited number of ASL studies that include the BS and those studies that do often use ASL techniques optimized for the whole brain,^9, 10^ rather than accounting for the local label kinetics of the BS. Furthermore, the presence of magnetic field inhomogeneities causes signal drop-out in regions near boundaries between air and tissue such as between the BS and the sphenoid sinus.

Another challenge of BS perfusion imaging with ASL is the relatively large contribution of intraarterial signal to the tissue perfusion signal.^11^ Note that the aim of ASL is to measure *tissue perfusion*, which means that the signal of interest arises from water that has passed into the brain parenchyma and not from labelled blood still present in the macrovasculature. Voxels in and around major arteries such as the basilar artery, which runs alongside the rostral aspect of the pons, contain a relatively large arterial blood volume (aBV). One approach to minimize the arterial signal is to apply bipolar gradients to reduce signal from moving spins.^12, 13, 14, 15^ This, however, will extend the echo-time (TE) increasing bulk susceptibility artifacts and reducing signal acquired. Furthermore, applying bipolar gradients means that it is impossible to measure aBV. Recently, modelling approaches to account for macrovascular signal have been developed.^16, 17^ The two-compartment model previously described by Chappell^16^ is used here.

The transit time of the tagged blood from the labelling location to the capillary bed, here referred to as *tissue arrival time* (TAT), constrains the time of image acquisition (postlabelling delay) required to ensure that labelled spins have entered the tissue of interest. The TAT varies across the brain^12, 15^ as does the *arterial arrival time* (AAT), the time it takes the blood to go from the tag location to the macrovascular bed in the region of interest (ROI).^12^ Because the kinetic curve of the magnetic label in the BS has not been fully investigated, both TAT and AAT have yet to be estimated for the BS. Their estimation would permit more efficient ASL data acquisition schemes in the future. Knowledge of the TAT in the BS, as for any brain region, would enable optimal timing of image acquisition, minimizing *T*_1_ decay of the label, especially useful when a restricted set of postlabelling delays or a single postlabelling delay is desired.

The aims of the present study were threefold. First, we improve signal acquisition in the BS using a short TE spiral acquisition resulting from pilot studies investigating image acquisition. Second, we characterize the kinetic curve of the magnetic label in the BS. From these data, we estimated BS perfusion parameters (i.e., CBF, TAT, aBV, and AAT). Tracking of the labelled blood over time was done by acquiring perfusion-weighted images at several delay times using multi-inversion time pulsed ASL (MTI PASL).^18^ Finally, we aimed to show the feasibility of MTI PASL to measure perfusion changes by performing a CO_2_ challenge to increase blood flow. This enabled assessment of the cerebral vascular reactivity (CVR) to hypercapnia (HC) in the BS, a measure of the vascular reserve.^19, 20^

## Materials and methods

Imaging data were acquired on a 3-T whole body MRI system (GE Excite HDx, Milwaukee, WI, USA) using an eight-channel receive-only head coil. Informed consent was obtained from all volunteers under ethical approval from the Cardiff University School of Psychology Ethics Committee and all experiments were performed in accordance with the guidelines stated in the Cardiff University Research Framework (version 4.0, 2010).

### Improving Signal Acquisition in the Brainstem

**Figure 1:**
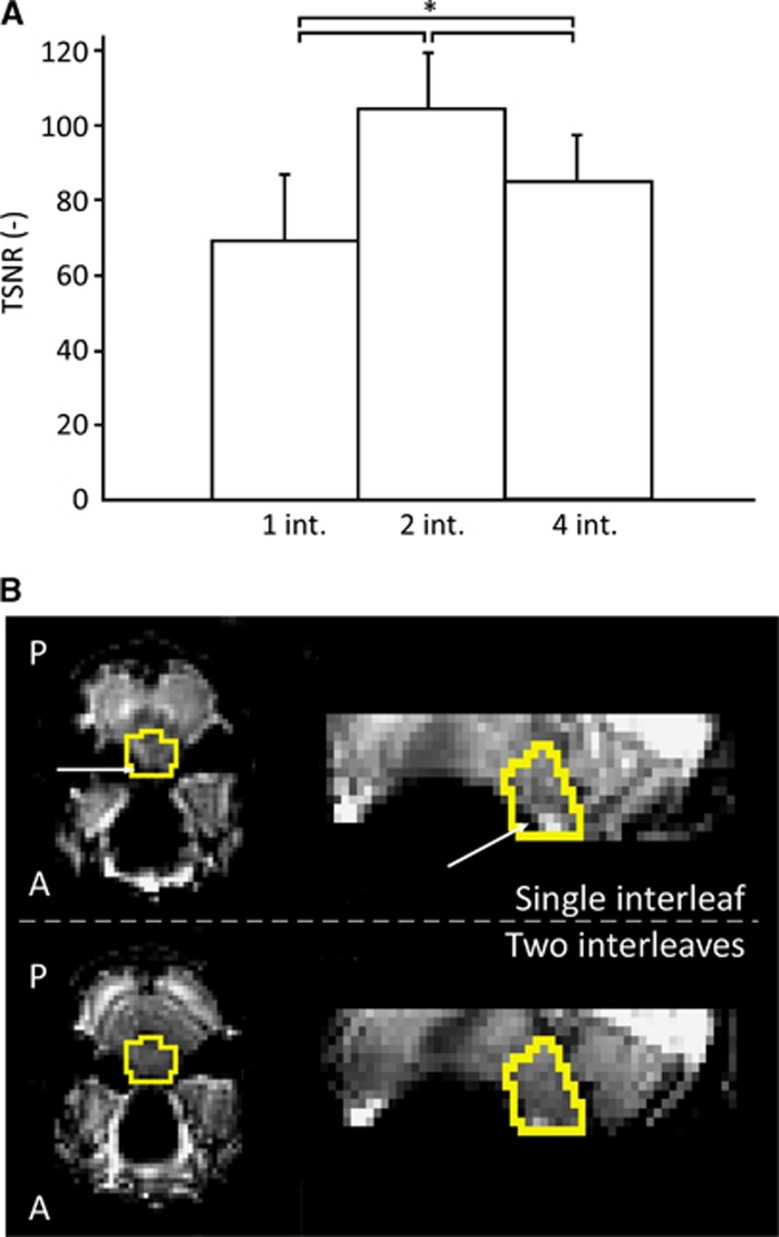
(**A**) Median temporal signal-to-noise ratio (TSNR) of the brainstem (BS) (group averages, *N*=5) for image acquisitions with 1, 2, and 4 spiral interleaves (int.). *Significantly different (pairwise comparison after repeated measures analysis of variance (RM-ANOVA), *P*<0.05). (**B**) Images from the pilot studies comparing acquisitions with a single spiral interleaf (top row) and two spiral interleaves (bottom row). BS outlined in yellow. Note the signal drop-out in the BS in the single spiral image (white arrows). The two interleaved spiral image has less distortion, because of a reduced acquisition window,^21^ shown by a more homogenous signal distribution in the BS and the better defined cerebellum.

Gradient-echo spiral-out image acquisition was implemented because spiral trajectories enable the use of short TE, leading to less dephasing effects and therefore improved signal recovery. We performed a pilot study with five healthy volunteers to investigate the use of multiple (2 and 4) spiral interleaves to reduce distortion in the BS.^21^ Three gradient-echo spiral-out series were acquired for each participant: (1) 100 images acquired with 1 interleaf, (2) 50 images acquired with 2 interleaves, and (3) 25 images acquired with 4 interleaves. Each series lasted 5 minutes. Results from this pilot study showed that using two interleaves leads to an increase in temporal signal to noise ratio (SNR) in the BS, calculated on a voxelwise basis as the mean signal over time divided by the temporal standard deviation of the signal. Pairwise comparison of median temporal SNR in the BS, after a repeated measures analysis of variance (RM-ANOVA) (*F*(2,8), *P*<0.001), showed that using two interleaves significantly improved temporal SNR in the BS compared with using a single interleaf (*P*<0.001). Examples of images acquired with one and two interleaves can also be seen in Figure 1. On the basis of the results of this pilot study, the acquisition parameters in the current study were as follows: 2 spiral interleaves, short echo (TE=2.7 ms), reconstructed matrix 64 × 64, voxel size=3 × 3 × 7 mm^3^, slice gap 1 mm, bandwidth 125 kHz, acquisition window 11 ms.

### Assessing the Kinetics of the Spin Label

#### Image acquisition

Seven young, healthy volunteers (3 female, mean age 29.6±4.9 years) were recruited to the initial study to investigate the kinetics of the label in the BS. Gradient-echo spiral imaging, as described above, was used with the following acquisition parameters: 2 interleaves, TE=2.7 ms, matrix=64 × 64, voxel size=3 × 3 × 7 mm^3^, slice gap=1 mm, 14 slices (whole brain coverage). Slices were tilted 10° to 15° from the axial to the coronal plane to reduce signal loss due to dephasing in the BS resulting from through-slice susceptibility-induced gradients.^22^ Automated linear shimming with the built-in software (GE HDx) was performed. Perfusion weighting was performed with MTI PASL^16^ (13 inversion times (TIs): 100 ms, 200 ms, 300 ms, 400 ms, 500 ms, 600 ms, 700 ms, 1,000 ms, 1,300 ms, 1,600 ms, 1,900 ms, 2,200 ms, and 2,500 ms). A PICORE tagging scheme was used with a QUIPSS II^23^ cutoff at 700 ms for TI>700 ms. Label thickness was 200 mm with a 10-mm gap between the distal end of the labelling slab and the most proximal imaging slice. A variable repetition time (1,000 ms to 3,400 ms) was used such that imaging time was minimized. Ten control–tag pairs were acquired for each inversion time, resulting in a total acquisition time of 18 minutes.

A calibration scan necessary for quantification of the perfusion maps was acquired in the beginning of each session to calculate the equilibrium magnetization of cerebrospinal fluid (*M*_0,CSF_). The acquisition parameters for this calibration scan were the same as for the perfusion-weighted scans, except for being acquired with fully relaxed magnetization and no labelling was applied. An image with minimal contrast was also acquired to map the coil sensitivity profile (same acquisition parameters except for TE=11 ms, repetition time=2 seconds, and 8 interleaves). Furthermore, a *T*_1_-weighted structural image (3D FSPGR) was acquired for registration purposes (TE=2.9 ms, repetition time=7.8 ms, voxel size=1 × 1 × 1 mm^3^).

#### Preprocessing

The time series of images was motion corrected using *3dvolreg* within AFNI (http://afni.nimh.nih.gov/afni^24^). Brain extraction was performed on the first image of the raw motion corrected series and the CSF calibration image with the brain extraction tool within the FMRIB Software Library v5.0 (FSL-http://fsl.fmrib.ox.ac.uk^25^). The CSF image was registered to the perfusion series using FLIRT within FSL and a mask of the lateral ventricles was used to calculate *M*_0,CSF_. This mask was made by first applying a threshold at 95% of the maximum signal intensity to the CSF image and then using AFNI's *3dclust* to find the largest cluster of voxels in a cube covering a large area of the brain, including both ventricles, in this masked image. The equilibrium magnetization for arterial blood (*M*_0,blood_) was then calculated according to methods previously described by Wong *et al*^23^ with CSF as a reference.

#### Kinetic curve analysis with the two-compartment model

**Figure 2:**
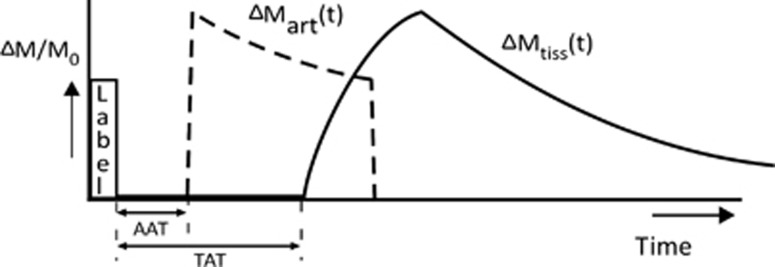
Schematic of Chappell's two compartment model.^16^ If a voxel has a significant macrovascular compartment, then an arterial model is fitted to the data (*ΔM*_art_), as well as a tissue perfusion model (*ΔM*_tiss_). The model also permits estimation of arterial arrival time (AAT) and tissue arrival time (TAT). Voxels without a significant macrovascular contribution will only have *ΔM*_tiss_ fitted to the data.

The unscaled difference images were created for each inversion time by subtracting consecutive tag and control images and averaging the result. Kinetic curves of the label were fitted on a voxel-by-voxel basis using Chappell's two-compartment model^16^ to separate tissue signal from arterial signal. In short, this model uses the following equation:


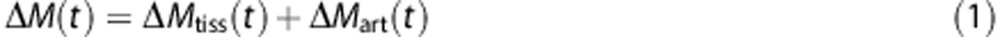


where *ΔM*_tiss_(*t*) is the signal in a perfusion-weighted difference image directly caused by tissue perfusion and therefore depends on CBF and TAT. *ΔM*_art_(*t*) is the signal intensity that is caused by magnetic label still present in the macrovasculature and therefore depends on aBV and AAT. *ΔM*_tiss_(*t*) is described by the general kinetic model for pulsed ASL, previously published by Buxton *et al.*^26^ The two-compartment model contains an automatic relevance determination algorithm that uses the perfusion-weighted data to do a voxelwise assessment of the aBV, which in turn is used to calculate *ΔM*_art_(*t*). If, as a result of the automatic relevance determination, aBV is set to 0, then equation (1) is reduced to *ΔM*(*t*)=*ΔM*_tiss_(*t*). A schematic representation of the model can be seen in Figure 2. Chappell's two-compartment model is incorporated as *oxford_asl* in the BASIL toolkit within FSL. The output of *oxford_asl* includes unscaled CBF maps, and maps of the estimated aBV, AAT, and TAT. In addition, maps containing the variance of each parameter estimate are calculated and can be used to calculate confidence intervals (CIs). Here, the unscaled CBF maps were scaled with *M*_0,blood_ to quantify CBF in mL/100 g per minute. Coil sensitivity correction was then performed by dividing the CBF maps by a normalized version of the minimal contrast image. Before normalization, the minimal contrast image was smoothed with an edge-preserving algorithm (SUSAN within FSL, full width at half maximum=5 mm).^27^

#### Registration and region of interest analysis

Region averages for CBF, TAT, aBV, and AAT were calculated for the following ROIs: whole BS (including medulla, pons, and the dorsal part of the midbrain), gray matter (GM, including cortical and subcortical GM), cerebellum (CB), and occipital pole (OP). The last two ROIs were added as a reference for the BS as it was either the nearest large brain structure at a similar rostral-caudal level (CB) or a distal brain structure (OP) for which longer arrival times would be expected.^15^ The Harvard Oxford atlas for cortical and subcortical structures, available within FSL, was used to obtain the five ROIs.

To minimize interpolation errors in the perfusion data due to registration, the region-based analysis was performed in individual subject space. Registration of the five ROIs from MNI152 standard space (Montreal Neurological Institute, Montreal, QC, Canada) to subject space was therefore performed. For this purpose, the brain extracted image of the motion corrected series (see *Preprocessing*) was registered to MNI152 standard space via the *T*_1_-weighted structural image. The resulting transformation matrix was inverted and applied to each of the five ROI masks.

All registrations were performed using FLIRT, within FSL. After region-based analysis, group average CBF and aBV maps were made by using the transformation matrix for registration of subject space maps to MNI152 standard space.

Within the analysis yielding aBV and AAT only voxels with a considerable arterial contribution (aBV>0.1%) were considered in the calculations of regional average aBV and AAT values. If an ROI on average did not have a large enough arterial contribution, aBV and AAT resulting from the two-compartment model were undefined and therefore not reported. If there was considerable aBV, average aBV is calculated by summing the aBV for the voxels with an aBV of >0.1% and dividing this by all the voxels in the ROI (including those voxels without arterial contribution). Average AAT is calculated by only considering the voxels within an ROI with measurable aBV.

### Hypercapnic Cerebral Vascular Reactivity

In general, the parameters for the HC experiment were kept the same as for the first experiment; however, the following paragraphs describe the differences in methods between the two experiments.

#### Image acquisition

Ten young, healthy volunteers (3 female, mean age 30.7±5.0 years) were scanned. Within each scan session, two perfusion scans were performed: one in normocapnia and one in HC, with the scan order randomized between subjects. All acquisition parameters for the perfusion scans remained the same for both conditions. The duration of the perfusion acquisition was reduced from 18 minutes used in the first experiment to ∼6 minutes to limit the duration of the hypercapnic challenge. Therefore, 6 inversion times (200 ms, 400 ms, 600 ms, 1,000 ms, 1,600 ms, and 2,200 ms) and 8 tag–control pairs per inversion time were acquired.

#### Hypercapnic respiratory challenge

Subjects breathed through a tight-fitting face-mask covering nose and mouth (Quadralite, Intersurgical, Wokingham, Berskhire, UK). Gases were delivered from gas cylinders connected to an in-house built and manually operated system of flow meters. Gases were humidified for the comfort of the participant. A gas mixing chamber, as close to the face mask as possible, had three feeding lines coming in for the delivery of medical air, 5% CO_2_, and medical oxygen, the latter incorporated as a safety backup but not used during experimentation.

During the *normocapnic* (NC) period participants breathed medical air (20.9% O_2_ balance N_2_) with a flow rate of 35 to 40 L/min. Baseline end-tidal CO_2_ pressure (P_ET_CO_2_) was determined at the start of each scan session, when participants were breathing medical air for ∼5 minutes. *Hypercapnia* was manually controlled by mixing medical air with 5% CO_2_ until P_ET_CO_2_ was raised by 7 to 8 mm Hg above individual participant's baseline. Two minutes of HC were established to stabilize P_ET_CO_2_ before image acquisition.

#### Physiologic monitoring

A sampling line connected to the face mask was used to monitor end-tidal CO_2_ and O_2_ concentrations. A respiratory belt was placed just below the ribs to monitor ventilation and a pulse oximeter finger cuff was used to obtain cardiac traces.

#### Cerebral vascular reactivity

In addition to the ROI analysis of CBF, TAT, aBV, and AAT as described above, the data analysis of the HC experiment included assessment of CVR to illustrate the feasibility of MTI PASL to measure changes in BS perfusion. The CVR is defined here as a change in tissue perfusion between NC and HC due to the change in P_ET_CO_2_ and is therefore reported as a percentage change in CBF per mm Hg (%/mm Hg).

#### Statistical analysis

Comparison of the physiologic parameters between different ROIs was performed with RM-ANOVA in SPSS Statistics for Windows, Version 20.0 (IBM Corp., Armonk, NY, USA) using *ROI* (i.e., BS, CB, OP, and GM) as the independent factor and CBF, aBV, AAT, and TAT as dependent factors. A two-way RM-ANOVA was performed with the data of the HC experiment in which *physiologic state* (i.e., NC or HC) was added as an independent factor. Follow-up pairwise comparisons were performed as appropriate using the Bonferroni correction. In some cases, the assumption of sphericity for the data did not hold (i.e., differences between ROI averages do not have equal variances). In those cases, the Greenhouse-Geiser correction for sphericity was used and this is reported by using *F*_corrGG_ instead of *F* in Results. Results are stated as *mean*±*standard deviation,* except where explicitly stated as *mean*±*standard error of the mean* to illustrate the confidence in the obtained mean values.

## Results

### Experiment 1: Label Kinetics

**Figure 3:**
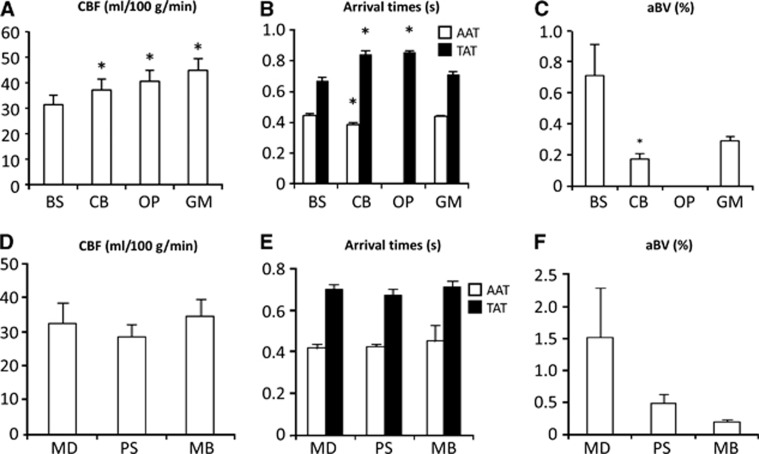
(**A**, **D**) Group mean cerebral blood flow (CBF) in mL/100 g per minute. (**B**, **E**) Arrival times in seconds. Note that no arterial arrival time (AAT) is reported for OP because this region of interest (ROI) did not have significant arterial blood volume (aBV) (explained in more detail in Materials and methods). (**C**, **F**) Group average aBV in %. Error bars in all bar charts reflect the standard error of the mean. BS, brainstem; CB, cerebellum; OP, occipital pole; GM, gray matter; MD, medulla, PS, pons; MB, midbrain. *Significantly different from the corresponding BS value (pairwise comparison after repeated measures analysis of variance (RM-ANOVA), *P*<0.05). TAT, tissue arrival time.

**Figure 4:**
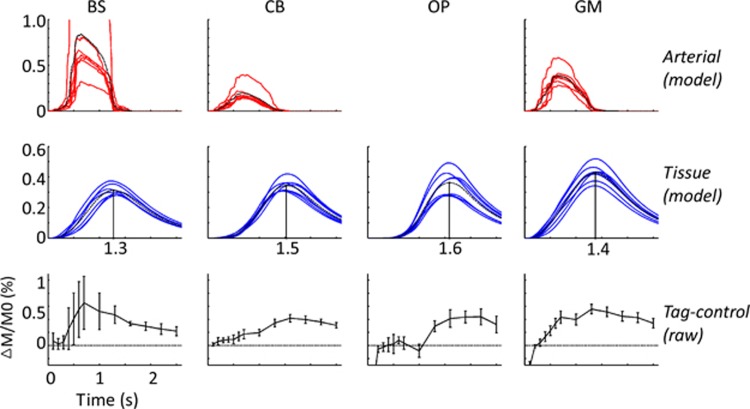
Regional average kinetic curves resulting from the model and raw difference signal for (from left to right) the brainstem (BS), cerebellum (CB), occipital pole (OP), and gray matter (GM) in the feasibility experiment (*N*=7). Top row: modelled kinetic curve of magnetic label in the macrovasculature. Individual kinetic curves in red and group averaged kinetic curve in dashed black. Middle row: modelled tissue perfusion curve (signal in microvasculature). Individual kinetic curves in blue and group averaged kinetic curves in dashed black. Bottom row: average signal in raw difference images, error bars indicate the standard deviation over seven subjects.

Repeated measures ANOVA (*F*(3,18)=8.6, *P*<0.01) and the follow-up pairwise comparisons showed that mean BS CBF (31.4±10.1 mL/100 g per minute, 68% CI 20.0 to 43.1 mL/100 g per minute) was significantly lower (*P*<0.05) than in GM (44.7±12.1 mL/100 g per minute, 68% CI 33.3 to 56.5 mL/100 g per minute). Tissue perfusion in CB and OP was also significantly different from that in the BS (*P*<0.05, Figure 3A). Group average tissue perfusion kinetic curves, calculated from the individual modelled kinetic curves (Supplementary Figure 1), can be seen in the second row of Figure 4. From these curves, it was calculated that maximum perfusion-weighted signal for the BS occurs 100 ms earlier than for the GM (1.3 seconds compared with 1.4 seconds, respectively).

Arterial arrival times for BS, CB, and GM were 444±31 ms (68% CI 341 to 551 ms), 384±37 ms (68% CI 268 to 503 ms), and 438±21 ms (68% CI 336 to 542 ms), respectively (see Figure 3B). Repeated measures ANOVA showed a significant difference in AAT between these ROIs (*F*_corrGG_(1,6)=7.9, *P*<0.05), with the CB having a significantly shorter AAT than the BS (*P*<0.05). No AAT is reported for the OP, as this ROI had no measurable aBV. Regional TATs do show significant differences between regions (see Figure 3B), with the BS having significantly shorter TAT than the CB and OP (pairwise comparisons after RM-ANOVA, *P*<0.001).

The modelled kinetic curves in Figure 4 show that the BS had the largest aBV of all the ROIs analyzed, which was also reflected by the high signal intensity at early TIs in the raw tag–control difference (bottom row in Figure 4). However, there was no significant difference (pairwise comparison after RM-ANOVA, *P*=0.063) between BS and GM aBV (see Figure 3C). Furthermore, the OP had no significant macrovascular contribution (average aBV<0.1%).

Repeated measures ANOVA showed that there were no significant differences in perfusion parameters between the subregions of the BS (medulla, pons, and midbrain in Figures 3D to F).

### Experiment 2: Hypercapnic Cerebral Vascular Reactivity

**Figure 5:**
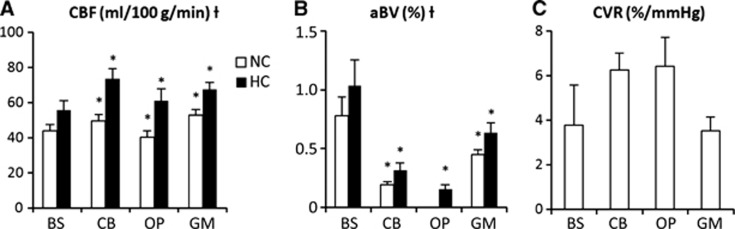
(**A**) Average cerebral blood flow (CBF) per region of interest (ROI) (mL/100 g per minute). All analyzed ROIs had significantly higher CBF in hypercapnia (HC) than in normocapnia (NC) (pairwise comparisons after repeated measures analysis of variance (RM-ANOVA), *P*<0.05). Furthermore, all analyzed ROIs had significantly higher CBF in both NC and HC than in the BS (pairwise comparisons after RM-ANOVA, *P*<0.05). (**B**) Average arterial blood volume (aBV) per ROI (%). Note that the BS had significantly higher aBV in NC and HC than in all other brain regions (pairwise comparisons after RM-ANOVA, *P*<0.05). The OP had no significant aBV in NC (aBV<0.1%), but did have significant aBV in HC. For all brain regions, the increase in aBV during HC was significant (pairwise comparisons after RM-ANOVA, *P*<0.05). (**C**) Group average cerebral vascular reactivity (CVR) per ROI (%/mm Hg). No significant differences were found between the CVR of the BS and the remainder of ROIs. Error bars in all three bar charts reflect the standard error of the mean. BS, brainstem; CB, cerebellum; OP, occipital pole; GM, gray matter. *Significant difference with the corresponding value for the BS (pairwise comparison after RM-ANOVA, *P*<0.05). ^†^Significant difference between values in NC and HC, over all ROIs (see text for details).

**Figure 6:**
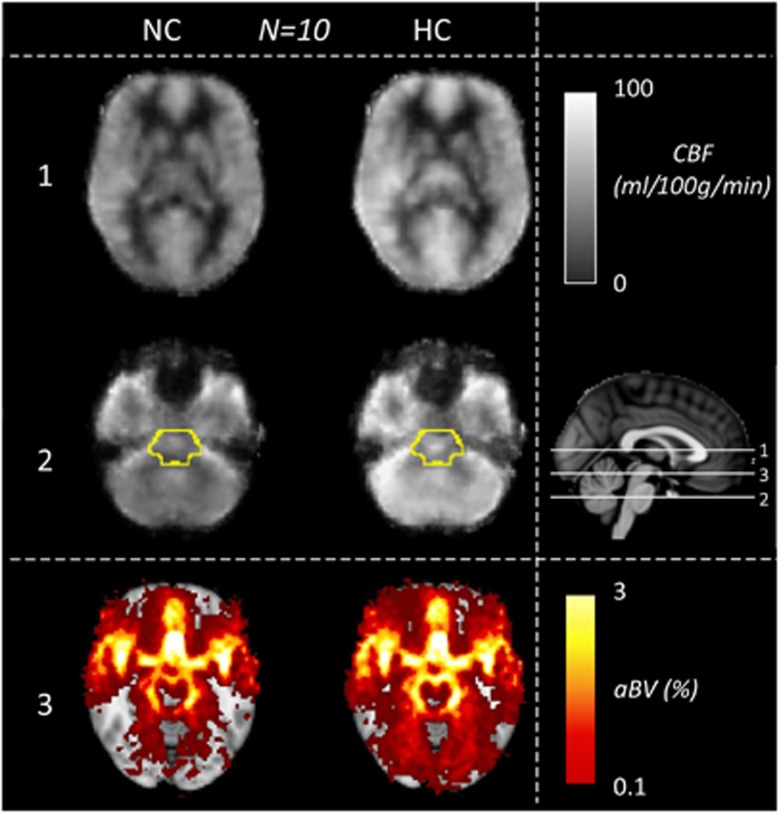
Group average perfusion maps (cerebral blood flow (CBF) in mL/100 g per minute) and arterial blood volume (aBV) maps (aBV in %) from the hypercapnia experiment (*N*=10). Maps are in Montreal Neurological Institute (MNI) standard space. The sagittal view on the right shows the locations of the axial slices on the left. The superior axial slice (slice 1) goes through the corpus callosum (top row). Slice 2 is an axial slice through the pons. In slice 2, the brainstem is outlined in yellow (mask from the Harvard Oxford subcortical atlas). Slice 3 (bottom row) is an axial slice through the midbrain, at the level of the Circle of Willis.

Baseline P_ET_CO_2_ was 39.4±2.4 mm Hg in NC and 47.3±1.9 in HC, a mean increase of 7.9±1.9 mm Hg (*N*=10). Two-way RM-ANOVA showed that the CBF significantly increased during HC (*F*(3,27)=12.0, *P*<0.001). Average GM perfusion (cortical and subcortical) was significantly higher than in the BS, both in NC and in HC (pairwise comparisons after RM-ANOVA, *P*<0.05). Brainstem CBF increased from 43.8±11.5 mL/100 g per minute in NC to 55.7±15.9 mL/100 g per minute in HC, while GM perfusion increased from 52.6±10.4 mL/100 g per minute in NC to 67.3±12.8 mL/100 g per minute in HC. Perfusion values for the remainder of ROIs can be seen in Figure 5A. Group mean perfusion maps resulting from the HC experiment are shown in Figure 6. The cerebral vascular responses for the BS (3.8±5.7%/mm Hg, s.e.m.=1.8%/mm Hg) and GM (3.5±1.9%/mm Hg, s.e.m.=0.6%/mm Hg) were not significantly different (RM-ANOVA, F_corrGG_(2,15)=1.95, *P*=0.180).

As expected, aBV showed significant increases during HC throughout the whole brain (RM-ANOVA, *F*_corrGG_(1,9)=14, *P*<0.01), as can be seen in Figure 5B and also illustrated by group average aBV maps (Figure 6). In both NC and HC, the BS had the highest aBV from all analyzed brain regions (pairwise comparisons after RM-ANOVA, *P*<0.01), as can be seen in Figure 5B. The OP had no significant aBV in NC (aBV<0.1%), but during HC the aBV increased to 0.16±0.1%. Group mean aBV maps can be seen in Figure 6 (bottom row), which shows how the aBV delineated cerebral macrovasculature (e.g., the Circle of Willis).

In the HC experiment, there were minimal differences in arrival times and modelled maxima of label kinetic curve, both between ROIs and physiologic states. Mean arrival times, both AAT and TAT, did show a trend toward a decrease in HC but this decrease was only significant for the TAT in the GM (pairwise comparison after RM-ANOVA, *P*<0.05).

## Discussion

Kinetic curves of the magnetic label in the BS can be obtained by acquiring perfusion images with MTI PASL and short-echo interleaved spiral read-out to yield CBF and arrival time estimates. Separating the macrovascular and microvascular signal using Chappell's two-compartment model was useful in the BS because of the high arterial contribution to the perfusion signal. This contribution is illustrated by the relatively large *ΔM*/*M*0 at TI<700 ms in the BS, as can be seen in the bottom row of Figure 4. Furthermore, the HC experiment showed that increases in BS perfusion can be measured with the current perfusion imaging and analysis method, as illustrated by the detected cerebral vascular response in the BS. To the best of our knowledge, this study is the first to use ASL to not only estimate CBF, but also assess CVR, TAT, AAT, and aBV in the BS of healthy participants.

### Brainstem Perfusion

Brainstem CBF was significantly lower than (cortical and subcortical) GM CBF. This may be expected since the BS consists of both gray and white matter in close proximity, the latter widely accepted as having lower perfusion.^28^ Previous studies also reported CBF lower in the BS than in global GM.^9, 29^ Furthermore, the BS CBF of ∼40 mL/100 g per minute reported in the HC experiment coincides with the CBF reported by Khalili-Mahani *et al*,^9^ who used pseudo continuous ASL (pCASL) to measure perfusion effects of alcohol and morphine on the whole brain and reported similar baseline BS CBF. In addition, Ito *et al*^29^ performed an HC experiment with PET and found similar BS CBF.

### Brainstem Cerebral Vascular Reactivity

The HC experiment showed the expected increases in CBF throughout the whole brain. In particular, the average GM CBF in HC (67.3±12.8 mL/100 g per minute at P_ET_CO_2_ of 47±1.9 mm Hg) coincides with the value found by Noth *et al*,^20^ who performed a similar hypercapnic experiment with a pulsed ASL perfusion measurement and found an average GM CBF of 71.3±5.9 mL/100 g per minute at P_ET_CO_2_ of 46.3±0.8 mm Hg. The average GM CVR of 3.5±1.9%/mm Hg is comparable to previously reported values measured with ASL,^19, 20, 30^ indicating that BS MTI PASL is able to measure CVR in GM.

Brainstem CVR values found here are similar to previously reported values measured with different imaging modalities.^29, 31^ Heistad *et al*^31^ used radio isotopes to measure vascular responses in anesthetized dogs and found similar CVR in pons and medulla: both ∼6%/mm Hg and 3.8±5.7%/mm Hg for the whole BS here. Ito *et al*^29^ reported a BS CVR of 11.4±14.9%/mm Hg measured with PET in eleven healthy men, which is almost three times larger than here. However, this might be explained by the surprisingly low reported increase in arterial CO_2_ pressure of only 3 mm Hg with a hypercapnic challenge using an inspired gas mixture containing 7% CO_2_. In comparison, here an average increase of almost 8 mm Hg in P_ET_CO_2_ is found with a gas mixture containing only 5% CO_2,_ which is comparable to other literature reports using similar respiratory challenges.^19^

Note that the intersubject variability of our measured BS CVR is relatively large (3.8 ±5.7%/mm Hg). Contributing to this is the low SNR of perfusion imaging in the BS. One solution for reducing the intersubject variability of BS CVR would be longer data acquisition, i.e., more than 8 tag–control pairs per inversion time, since analysis of our data with just 4 tag–control pairs resulted in an increase in intersubject variability of ∼70% (*data not shown*).

### Arrival Times

Arterial arrival times resulting from the first experiment are higher than previously reported values (>400 ms here vs. <400 ms elsewhere^12, 14^), which could be explained by the use of different pulsed ASL techniques. Here, the arterial compartment of the model contains an almost instantaneously increasing slope (see *ΔM*_art_(*t*) in Figure 2), leading to a rapid inflow of labelled blood being modelled. This effect results in the AAT depending strongly on the first TI where signal intensity in the unscaled difference images is larger than zero (TI=400 ms, bottom row in Figure 4). Reported methods for measuring AAT differ, for example, Chen *et al*^12^ used flow-sensitive alternating inversion recovery, a pulsed ASL method that does not require a gap between the label and imaging plane and Ho *et al*^14^ report AAT within the visual cortex (OP) based on a single slice for which the labelling plane was positioned closer to the OP than in the current study. In both these studies, the label travelled a smaller distance before arriving at the imaging plane, likely explaining the shorter AATs.

Tissue arrival times as calculated from the kinetic curve experiment compare favorably with previously reported TAT in MTI PASL experiments.^12, 13, 14, 18, 32^ The difference in TAT between proximal and distal brain regions is comparable (250 to 300 ms in all six studies, including the current experiment) and is also reflected by the maxima in modelled tissue perfusion curves (1.3 seconds in BS and 1.6 seconds in OP, Figure 4). In the literature, TATs range from 500 ms to 950 ms,^12, 13, 14, 18, 32^ which also coincides with the values reported here. On average, studies including additional gradients to crush flow in large arteries have slightly shorter average arrival times for proximal ROIs,^12, 13, 32^ which might be caused by the flow crusher gradients not completely removing arterial signal.^16^

The BS AAT (444±31 ms) and TAT (670±51 ms) found in the kinetic curve experiment coincide with the data in the unscaled tag–control images, as can be seen by comparing the modelled arterial and tissue kinetic curves with the tag–control curve in Figure 4, giving face validity to the two-compartment model in the BS. In combination with the shorter time to maximum perfusion signal in the BS compared with GM (1.3 seconds in the BS and 1.4 seconds in the GM in Figure 4), the arrival time findings imply that for future single inversion time ASL experiments concerning the BS it could offer more rapid imaging and a higher SNR to use a shorter inversion time than for whole brain ASL imaging.

The use of only six inversion times, with gaps of ⩾200 ms between TIs, in the measurement of hypercapnic CVR resulted in lower sensitivity to differences in arrival times. This led to less measured interregional dispersion and limited detection of decreased arrival times in HC, which are expected to be ⩽100 ms shorter than in NC (based on previous results by Ho *et al*^33^). Nevertheless, we did observe a significant decrease in TAT (but not in AAT) during HC in GM. This finding suggests that changes in arterial and TATs due to a functional challenge (i.e., HC) might (partly) have separate mechanisms. This suggestion has also been made by Ho *et al*,^14^ who found that both AAT and TAT decreased in the visual cortex during visual stimulation, but while the decrease in AAT was constant with different stimulation strength the TAT decreased more with stronger stimulation. To thoroughly investigate this ‘uncoupling' of arrival times for HC, we would suggest an MTI PASL experiment with more than six inversion times.

### Physiologic Noise

The choice of interleaved spiral acquisition adds certain difficulties to address the problem of physiologic noise in the BS. Respiratory motion and cardiac pulsation are two important sources of physiologic noise in BS imaging in addition to those mentioned in Introduction.^34^ Established methods exist to reduce this physiologic noise retrospectively from the imaging data such as RETROICOR,^35^ which determines cardiac and respiratory phases per image slice and regresses these components from the MR time series. Here, the use of RETROICOR is confounded because two consecutive repetition times are used to create a single image.

However, the use of two interleaves in itself decreases the presence of physiologic motion because of the fact that one image is a combination of measurements at two time points, each acquired with a k-space trajectory starting at the k-space origin (the same spiral, but shifted 180°). As a result, an interleaved spiral acquisition would be expected to be less sensitive to low-frequency physiologic noise than a single spiral acquisition.^21^

### Signal to Noise

It would be desirable to increase image resolution, both in-plane and through-plane given the small structures that are of biologic interest in the BS.^1, 2^ The voxel sizes used here (63 mm^3^) do not enable cerebrovascular assessment of individual BS nuclei, most of them with diameters<1 mm.^1^ The current data do allow investigation of subregions in the BS (i.e., medulla, pons, and midbrain). However, no significant differences between these regions were found in perfusion or arrival times so far (Figure 3). Not finding significant differences between these regions, even in arrival times, is partly explained by the fact that the subregions are small, decreasing the already low SNR even further. Increasing SNR of pulsed ASL, for example by imaging at stronger field strengths (e.g., 7 T) or adding denoising methods, could permit perfusion-weighted imaging with higher resolution.

Previous studies have shown up to threefold increases in SNR of ASL images by using 3D acquisition methods such as 3D GRASE and 3D FSE spiral acquisitions.^36^ Brainstem ASL might especially benefit from the latter, because of reduced sensitivity to magnetic susceptibility variations compared with 3D GRASE.^36^ However, due to software limitations it was not possible to include a 3D FSE spiral acquisition in the current study.

Another possibility for increasing SNR in perfusion imaging would be to use pCASL instead of pulsed ASL, as under ideal circumstances the SNR of pCASL is higher than for PASL due to the extend duration of the labelling.^37^ However, a limitation of pCASL is that the labelling efficiency decreases with increasing blood velocity risking the underestimation of increases in CBF.^38^ Therefore, particularly in studies where changes in blood flow are to be expected, such as evaluation of CVR using HC, caution needs to be taken with interpreting the results from pCASL images. However, similar caveats must be considered with PASL. A large spatial bolus width in PASL, such as used here, aims to desensitize PASL methods to blood velocity by ensuring that the trailing edge of the bolus is subject to a saturation pulse at the cutoff time.^23^ However, if in this case blood velocities are larger than 29 cm/s (label width 200 mm, cutoff at 700 ms) the current PASL method would also underestimate CBF in HC. Because average blood velocities in the vertebral and internal carotid arteries in healthy volunteers have been reported to be 22 and 29 cm/s, respectively, at a similar P_ET_CO_2_ level in HC as used here,^39^ there is some risk of underestimating CBF and CVR in the current study arising in particular from the blood flow velocity in the carotid arteries.

### Applications of Brainstem Perfusion Measurements

As mentioned in Introduction possible clinical application of measures for BS vascular function is to aid diagnosis and assessment of treatment of pathologies. Measurement of brainstem CBF and CVR with pulsed ASL as introduced here has the advantage over PET of being noninvasive and easily applicable in the clinic because there is no need for on-site production of radioactive tracers. A drawback of the current multi-inversion time ASL method for clinical application is the relatively long imaging time (e.g., 20 minutes to obtain 13 inversion times). However, the estimation of BS arrival times enables design of single inversion time perfusion imaging specifically for the BS and although having a single TI prevents explicit arrival time measurements, it will enable CBF and CVR measurements with considerably shorter acquisition time.

In contrast with ASL, blood oxygen level-dependent imaging is limited to measuring transient responses to a (functional or physiologic) task which are dependent on neural activation and baseline hemodynamics.^40^ Without knowledge of this baseline state, comparison of blood oxygen level-dependent activation maps between different physiologic, for example, drugs or disease, states is rendered difficult.^2, 40^ Therefore, in addition to giving a direct measure of BS physiology, the current perfusion methods would also complement BS blood oxygen level-dependent imaging studies.

Brainstem MTI PASL has resulted in robust estimation of the kinetic curve of the magnetic label in the BS. The outcome of this study includes estimates of plausible values for BS perfusion and, arterial and tissue arrival times. Furthermore, MTI PASL for BS perfusion imaging was validated by measurement of increased perfusion during HC, resulting in an estimate of CVR in this brain region. These measures of BS cerebrovascular function have the potential to be of use in diagnosis and follow-up of BS-related pathologies and can be used to complement blood oxygen level-dependent imaging.
