# Supplementary figures and images for: In vivo assessment of human brainstem cerebrovascular function: a multi-inversion time pulsed arterial spin labelling study
Source: J Cereb Blood Flow Metab. 2014 Mar 5;34(6):956–63. doi: 10.1038/jcbfm.2014.39 (PMC4050237; doi:10.1038/jcbfm.2014.39)

# Average signal per region ( $\Delta M/M_0$ in %)

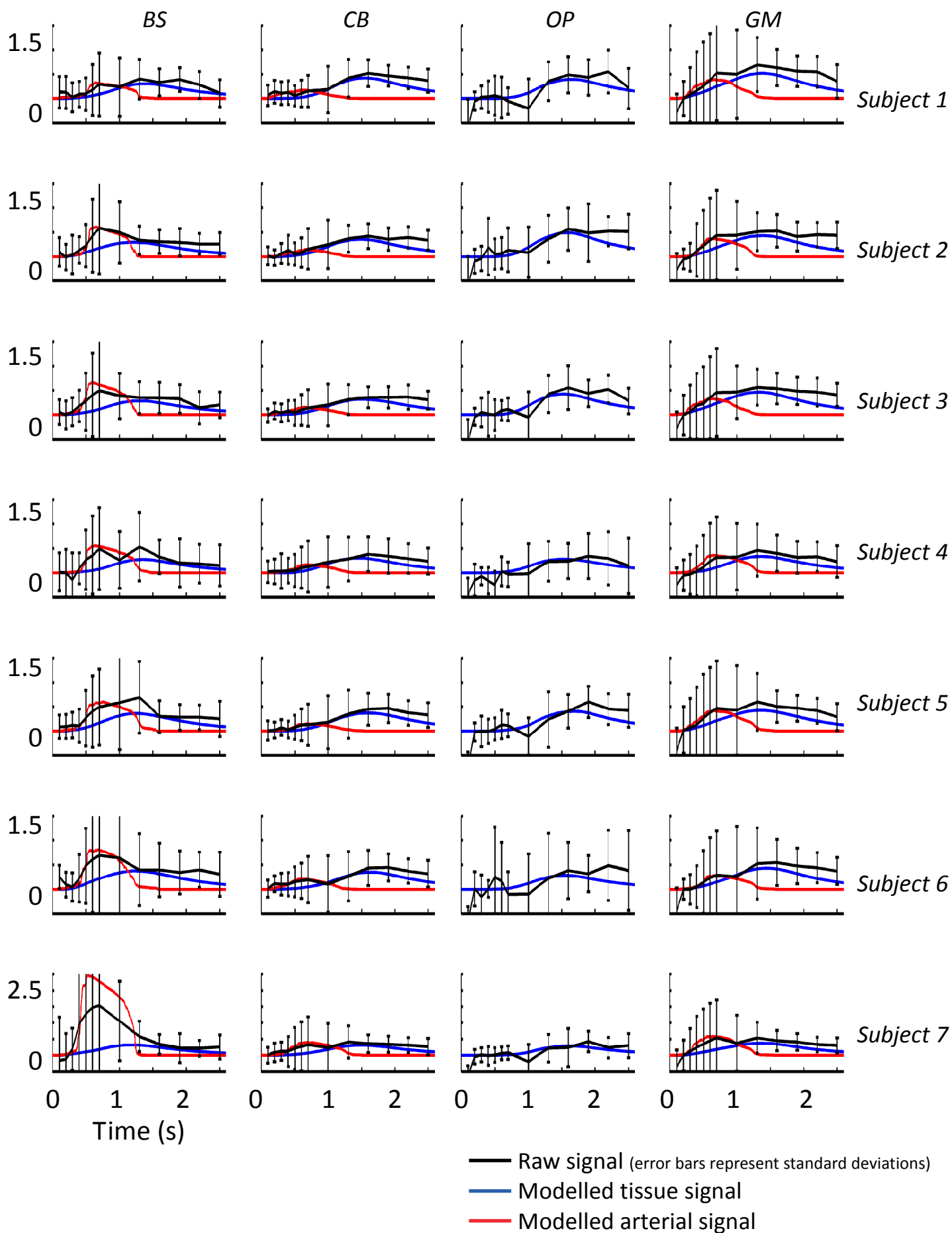

Supplement: Supplementary Figure 1 [file jcbfm201439x1.pdf]
