# Supplementary material for: In vivo assessment of human brainstem cerebrovascular function: a multi-inversion time pulsed arterial spin labelling study
Source: J Cereb Blood Flow Metab. 2014 Mar 5;34(6):956–63. doi: 10.1038/jcbfm.2014.39 (PMC4050237; doi:10.1038/jcbfm.2014.39)
Supplement: Supplementary Figure Legend [file jcbfm201439x2.doc]

Supplementary Figure 1. Individual kinetic curves resulting from *Experiment 1: Label kinetics*. Kinetic curves are shown per ROI (*BS* = brainstem, *CB* = cerebellum, *OP* = occipital pole, *GM* = gray matter). The average tag-control difference signal is printed in black, with the error bars representing one standard deviation. The kinetic curves resulting from fitting the two-compartment model to the tag-control differences are printed in red (macrovascular or arterial signal) and blue (microvascular or tissue signal).
